# A story from the Miocene: Clock‐dated phylogeny of *Sisymbrium* L. (Sisymbrieae, Brassicaceae)

**DOI:** 10.1002/ece3.7217

**Published:** 2021-03-02

**Authors:** Anže Žerdoner Čalasan, Dmitry A. German, Herbert Hurka, Barbara Neuffer

**Affiliations:** ^1^ Department 5: Biology/Chemistry, Botany University of Osnabrueck Osnabrueck Germany; ^2^ South‐Siberian Botanical Garden Altai State University Barnaul Russia

**Keywords:** Brassicaceae, dated phylogeny, Eurasian steppe, florogenesis, *Sisymbrium*, taxonomy

## Abstract

Morphological variability and imprecise generic boundaries have hindered systematic, taxonomical, and nomenclatural studies of *Sisymbrium* L. (Brassicaceae, Sisymbrieae DC.). The members of this almost exclusively Old‐World genus grow mostly on highly porous substrates across open steppe, semidesert, or ruderal habitats in the temperate zone of the Northern Hemisphere and African subtropics. The present study placed the biological history of *Sisymbrium* L. into time and space and rendered the tribus Sisymbrieae as monotypic. Five nuclear‐encoded and three chloroplast‐encoded loci of approximately 85% of all currently accepted species were investigated. Several accessions per species covering their whole distribution range allowed for a more representative assessment of intraspecific genetic diversity. In the light of fossil absence, the impact of different secondary calibration methods and taxon sets on time spans was tested, and we showed that such a combinatorial nested dating approach is beneficial. Multigene phylogeny accompanied with a time divergence estimation analysis placed the onset and development of this tribus into the western Irano‐Turanian floristic region during the Miocene. Continuous increase in continentality and decrease in temperatures promoted the diversity of the Sisymbrieae, which invaded the open grasslands habitats in Eurasia, Mediterranean, and South Africa throughout the Pliocene and Pleistocene. Our results support the assumption of the Irano‐Turanian region as a biodiversity reservoir for adjacent regions.

## Graphical abstract

Morphological variability and imprecise generic boundaries have hindered systematic, taxonomical, and nomenclatural studies of *Sisymbrium* L. (Brassicaceae, Sisymbrieae DC.). The present study placed the biological history of *Sisymbrium* L. into time and space and rendered the tribus Sisymbrieae as monotypic. It appears that palaeogeographical and palaeoenvironmental events during the Pliocene and Pleistocene are the main driving forces behind the evolutionary history of many Sisymbrieae species.

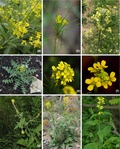

## 1 INTRODUCTION

*Sisymbrium* L. (Brassicaceae, Sisymbrieae DC.) belongs to one of the most well‐studied and economically important plant families in the world, including important vegetables and forage crops, condiments, decorative plants, and model organisms. Nevertheless, morphological homogeneity within the genus on the one hand and extreme intraspecific morphological variability and lack of precise generic boundaries on the other hand have hindered systematic studies of *Sisymbrium* until the last decade (Al‐Shehbaz, 2006b; Warwick et al., 2002, 2006). Much effort has been put into elucidating the actual number of *Sisymbrium* species, starting with the first worldwide monographic study by Fournier as early as 1865. His generic concept, however, was based very broadly and is nowadays considered outdated (Warwick et al., 2006). Several other taxonomic studies on *Sisymbrium* followed including Payson (1922) and Schulz (1924, 1928). Schulz's circumscription of the genus was based on nonapomorphic single characters only, including linear and terete fruits (siliques) and seeds with incumbent cotyledons. Thus, it was criticized in subsequent studies on *Sisymbrium* by Rollins (1943) and Romanczuk (1981, 1982). It was Rollins (1982) who first regarded *Sisymbrium* as an exclusively Old World taxon. Nevertheless, under current circumscription *Sisymbrium* indeed includes one single non‐Eurasian species, *Sisymbrium linifolium* Nutt. Its distribution area was believed to be limited to the dry habitats of the western USA, but it was also recently discovered in north‐western China (Chen et al., 2019). While being highly controversial in the past, the generic limits of *Sisymbrium* are nowadays well‐understood and generally accepted (following Al‐Shehbaz, 2012, 2015; Rollins, 1982) with a notable exception of *Sisymbrium aculeolatum* Boiss. and *Sisymbrium afghanicum* Gilli (Warwick & Al‐Shehbaz, 2003). While these two taxa have been formally moved from *Sisymbrium* to *Neotorularia* Hedge & Léonard (Léonard, 1986), there was only little justification for such a transfer (Appel & Al‐Shehbaz, 2002). Furthermore, the whole plastome phylogeny placed *S. aculeolatum* next to Sisymbrieae (Walden et al., 2020). Hitherto it remains unclear, whether the two aforementioned species belong to the genus *Sisymbrium* or not.

Together with the monotypic *Ochthodium* (Nikolov et al., 2019; Walden et al., 2020) *Sisymbrium* belongs to the tribus Sisymbrieae, which shows a close relationship with the other tribes in the Lineage II of the Brassicaceae phylogeny—Brassiceae, Isatideae, and Thelypodieae (BrassiBase: Kiefer et al., 2014; Koch et al., 2012, 2018). Currently, there is no consent either which of these tribes are sister group to the Sisymbrieae, or which of the other clades build a sister group relationship within the Lineage II (BrassiBase: https://brassibase.cos.uni‐heidelberg.de). A number of single, multi‐locus, and whole‐genome studies recovered contradicting sister group relationships with Sisymbrieae (Al‐Shehbaz et al., 2006; Beilstein et al., 2010; Couvreur et al., 2010; Franzke et al., 2011; German et al., 2009; Huang et al., 2016, 2020; Liu et al., 2020; Nikolov et al., 2019; Walden et al., 2020; Warwick et al., 2010).

Warwick et al. (2002) published the last and the most comprehensive phylogeny of *Sisymbrium* L. based on nuclear genes, which also served as an anchor point for our study. However, over the past two decades, new species of *Sisymbrium* have been discovered and validly described (Al‐Shehbaz, 2015; Blanca et al., 2015; Mutlu & Karakuş, 2015), several taxonomic revisions took place (Al‐Shehbaz, 2004a, 2004b, 2006a, 2006b; German & Al‐Shehbaz, 2018; Warwick & Al‐Shehbaz, 2003), and the shortcomings of the use of single potentially paralogue markers have been uncovered (Koch et al., 2007; Pirie et al., 2007). No comprehensive chloroplast‐based *Sisymbrium* phylogeny has been published until now. Furthermore, only scarce genetic data of individual chloroplast regions (Arias & Pires, 2012; Hall et al., 2002; Koch et al., 2007; Warwick et al., 2004, 2006) or whole plastomes (Hohmann et al., 2015; Nikolov et al., 2019; Walden et al., 2020) are available, preventing a comprehensive comparative study. Out of 514 different *Sisymbrium* names that can be found in the literature, currently only 50 of them represent accepted species and subspecies, leaving out 466 synonyms and one name with an unresolved status (Kiefer et al., 2014; Koch et al., 2018).

All *Sisymbrium* representatives exhibit incumbent cotyledons; often pinnately divided never clasping leaves; indumentum, when developed, of simple trichomes (with the only exception of South African *S. burchellii*, having branched trichomes—Marais, 1970); yellow (or rarely white or pinkish) flowers; two‐lobed stigmas; cylindrical fruits with three‐veined valves, and seeds that are arranged in a single row (Al‐Shehbaz, 2015, Figure 1). In terms of chromosome number, *Sisymbrium* is rather monomorph with *x* = 7, seldom *x* = 8 (Al‐Shehbaz, 2015). Most of the species exhibit only one ploidy level, that is, diploid with occasional tetraploid exceptions, such as *Sisymbrium luteum*, *Sisymbrium polyceratium,* and *Sisymbrium strictissumum* (all: 2*n* = 4*x* = 28) or hexaploid *Sisymbrium runcinatum* (Al‐Shehbaz, 1988). *Sisymbrium irio* is the most diverse in terms of ploidy (Khoshoo, 1959). While it is mostly diploid throughout its native and naturalized ranges of temperate Eurasia, Mediterranean region, and both Americas, tetraploids, hexaploids, and octoploids have been reported from the Afghanistan and Indian subcontinent (Khoshoo, 1959; Podlech & Dieterle, 1969). Most of the weedy species are self‐compatible and autogamous. Some species such as *Sisymbrium irio*, *S. officinale,* and *S. orientale*, however, develop self‐compatible protogynous flowers (Al‐Shehbaz, 1977; Khoshoo, 1959). *Sisymbrium* is characterized by variable glucosinolate profiles and presence of cardioactive steroid glycosides (cardenolides), which are otherwise extremely scarce in the Brassicaceae (Al‐Shehbaz, 1988; Fahey et al., 2001). One of notable exceptions to this rule is the genus *Erysimum* (Züst et al., 2020), which superficially morphologically resembles *Sisymbrium*. A number of medicinal properties have been attributed to several *Sisymbrium* species (one of the most prominent examples is *S. officinale*, hence the name), due to their analgesic, antibacterial, diuretic, and myorelaxant activity (Al‐Shehbaz, 1988; Blažević et al., 2010; Di Sotto et al., 2010).

**FIGURE 1:**
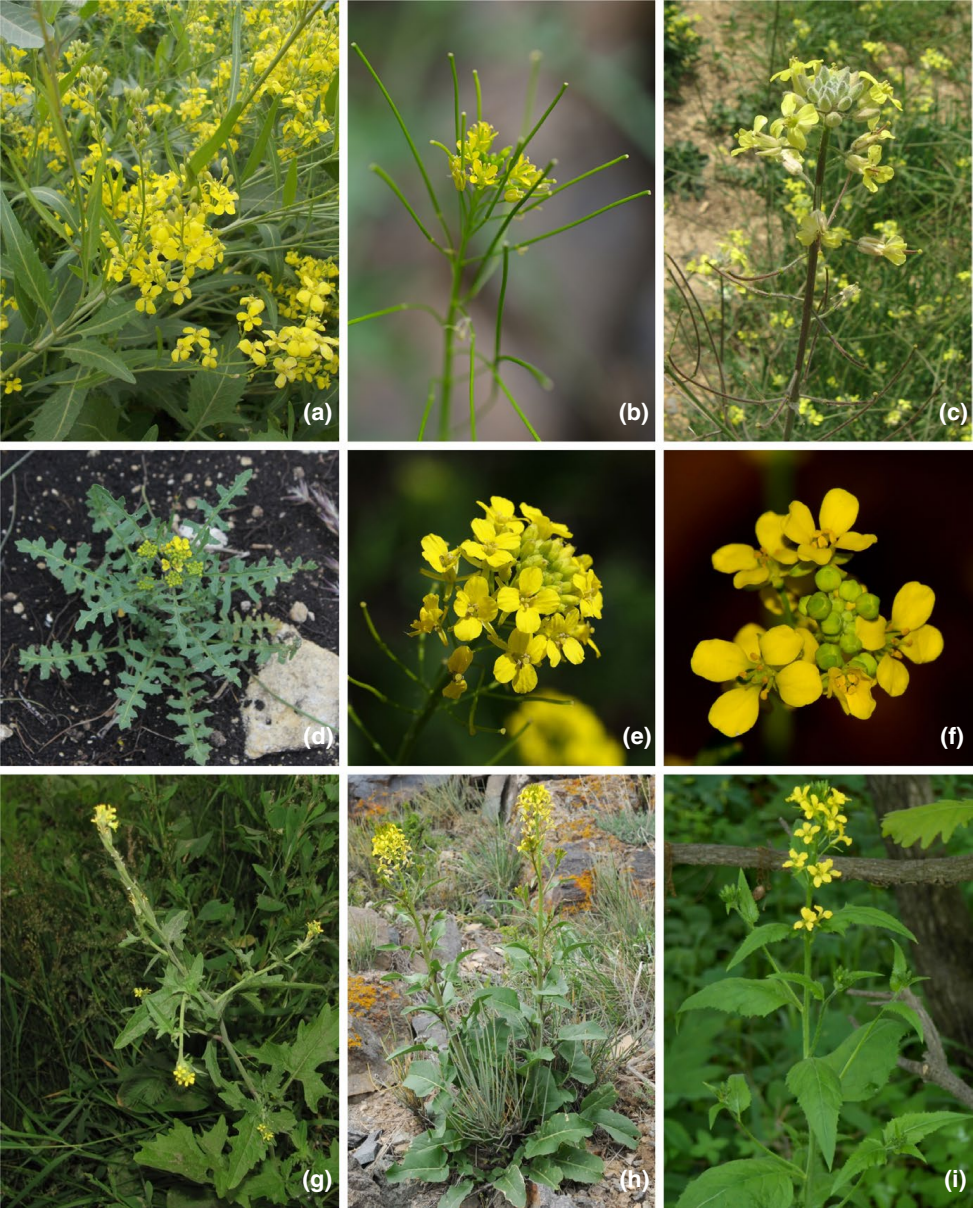
Habitus of different Sisymbrieae representatives. a – *Sisymbrium volgense* (Irkutsk region; June 27th 2012; by A. Manchenko), b – *Sisymbrium heteromallum* (Mongolia, Khovd aimak, Jargalant‐Khairkhan; June 25th 2015; by P. Kosachev), c – *Sisymbrium orientale* (Ukraine, Crimea; May 17th 2013; by A. Fateryga), d – *Sisymbrium lipskyi* (Karachay‐Cherkesskia, Malokarachaevsky district; June 15th 2013; by I. Tabunova), e – *Sisymbrium loeselii* (Krasnodar Territory, Novorossiysk, Abrau‐Dyurso; June 3rd 2009; by V. Gumenyuk), f – *Ochthodium aegyptiacum* (Israel, Lower Galilee, Upper Nazareth; March 25th 2014; author of the photography wanted to remain anonymous), g – *Sisymbrium officinale* (Yaroslavl; June 24th 2012; by E. Zakharov), h – *Sisymbrium brassiciforme* (Kazakhstan, Kapchagai; April 17th 2019; by V. Epiktetov), i – *Sisymbrium luteum* (Primorsky Territory, Nakhodka; June 14th 2012; by V. Volcotrub)

The center of *Sisymbrium* diversity is the Irano‐Turanian region (sensu Takhtajan et al., 1986), followed by temperate regions of Europe and the Mediterranean (http://www.plantsoftheworldonline.org/). Only a handful of species can be found in non‐Mediterranean North as well as in South Africa and a single already above‐mentioned species grows in the dry habitats of the western USA and Canada. Despite showing a variation of lifestyles and distinct morphological features in the vegetative as well as in reproductive organs, most *Sisymbrium* species can be found in or near dry open steppe habitats where they grow on limestone, sand or gravel substrates, or in semideserts in the temperate zone of the northern hemisphere and African subtropics. However, some species such as *Sisymbrium altissimum*, *S. irio*, *S. loeselii*, *S. officinale,* and *S. volgense* are known for being successful ruderal‐occupying alien species (Jehlík, 1981; Mito & Uesugi, 2004; Oprea & Sîrbu, 2010; Protopopova et al., 2006; Weber et al., 2008) that have spread throughout Europe or Asia from their native habitats in the East European Plain and western Irano‐Turanian region.

The southern East European Plain is part of a nowadays continuous Eurasian steppe belt, which is the vastest grassland region in the world with 8,000 km in length and up to 1,000 km in width. This ecoregion was under strong influence of glacial and interglacial cycles during the past five MYR, which continuously caused contractions, expansions, and latitudinal range shifts of the Eurasian steppe belt (Hurka et al., 2019). In contrast, the western and central regional subcenters of the Irano‐Turanian region (Léonard, 1988) were not as strongly affected by the Quaternary glaciations as the Eurasian steppe belt. Only intermountanious valley glaciations were recorded from the Pleistocene cycles, leaving ancestral flora at least partially intact (Agakhanjanz & Breckle, 1995). This is corroborated also by high levels of endemic species in Iranian mountain ranges and current distribution patterns of several representative Irano‐Turanian plant taxa (Djamali, Baumel, et al., 2012; Djamali et al., 2012; Noroozi et al., 2008). The western and central regional subcenters of the Irano‐Turanian region may have played an important role by partially “supplying” adjacent regions—northern and eastern Irano‐Turanian subcenters, as well as the Mediterranean Basin and Saharo‐Arabian floristic region—with floral elements (Magyari et al., 2008; Manafzadeh et al., 2014, 2017; Takhtajan et al., 1986; Žerdoner Čalasan et al., 2019).

The overall aim of our studies is to place the biological history of *Sisymbrium* L. into time and space. To achieve that we carried out a multigene phylogeny using biparental fast‐evolving ITS and ETS regions, as well as low copy nuclear markers Bra13, Bra246, Bra813, Bra1402, and maternal chloroplast markers trnQ‐rps16, psbA‐trnH, and ycfb1 of a representative taxon set, including approximately 85% of all currently recognized species. Due to fossil absence, we further tested the impact of different tree priors, calibration methods (substitution rates vs. secondary calibration points), and different taxon sets on the topology as well as time spans of the Sisymbrieae clade. In addition, aiming for taxonomic stability, we also formally transferred *Ochthodium aegyptiacum* DC. to *Sisymbrium* and investigated the phylogenetic position of *Sisymbirum aculeolatum* and *S. afghanicum*. To achieve the latter and test the robustness of our time estimation, we extended the phylogenetic analysis and time divergence estimation to the whole Lineage II.

## 2 MATERIALS AND METHODS

### 2.1 Taxon sampling and distribution range surveys

We compiled a taxon sample covering the whole distribution area of all collected species of *Sisymbrium*. Distribution maps were put together primarily using geographical range information from the literature (Brach & Song, 2006; Grubov, 2001; Jalas & Suominen, 1994; Malyschev & Peschkova, 2004; Meusel et al., 1965; Post, 1896; Tutin et al., 1993; Vassilczenko, 1939) and our own field data, and only secondarily using online databases (Euro + med PlantBase: http://ww2.bgbm.org/EuroPlusMed/query.asp; GBIF: https://www.gbif.org; Flora of Mongolia: https://floragreif.uni‐greifswald.de/category/literature/; Plantarium: https://www.plantarium.ru/; Plants of the World Online: http://www.plantsoftheworldonline.org/; Weeds of Australia : shorturl.at/aduzS), whose entries were critically assessed prior inclusion in the survey. Plant material was obtained from specimens deposited in B, MSB, GDA, GDAC, HBG, HEID, HUJ, K, L, P, MW, PRE, OSBU, and WAG herbaria (Appendix S1). Geographical data were recorded at the specimen collection site (in the case of our own collection), recovered directly from the voucher specimens, or reconstructed by using TopGlobus (http://www.topglobus.ru/), Wikimapia (http://www.wikimapia.org/), and Loadmap (http://loadmap.net/) based on text information from the voucher specimens. The maps for ancestral range reconstruction were generated with QGIS (https://www.qgis.org/en/site/), and all the figures were adjusted in Inkscape (https://inkscape.org).

### 2.2 Molecular analyses

Total genomic DNA was isolated from herbarium specimens using the InnuPREP Plant DNA Kit (Analytic Jena AG) according to the instructions of the manufacturer and used directly in PCR amplifications. PCR was carried out using 10 µl of 2× HS Taq Mix Red (Biozym Scientific), 7 µl of ddH_2_O, 1 µl of each 10 µM primer, and 1 µl of template DNA. Alternatively, PCR was carried out using 20.8 µl of ddH_2_O, 3 µl of 10× Taq Buffer with MgCl_2_, 2 µl of 10mM dNTP mixture, 1 µl of DMSO, 0.2 µl of 5U Taq Polymerase, 1 µl of corresponding primers, and 1 µl of the template DNA. We analyzed the following nuclear‐encoded loci: internal transcribed spacer, including the intervening 5.8S region (ITS1‐5.8S‐ITS2), external transcribed spacer (ETS), Bra264 exon and Bra13, Bra813 and Bra1402 loci all comprised of two introns and three exons (Stockenhuber et al., 2015). Furthermore, three additional chloroplast regions were investigated: trnQ‐rps16 intergenic spacer, psbA‐trnH intergenic spacer, and ycf1b coding locus. Used primers and PCR programmes can be inferred from Appendix S2. Samples that yielded single bands once run through gel electrophoresis were sent to Microsynth Seqlab (Goettingen, www.microsynth.ch) for purification and sequencing. Sequences from each accession were manually edited in Chromas Lite 2.6.4 (Chromas | Technelysium Pty Ltd) and aligned and manually corrected in AliView v1.24 (Larsson, 2014).

### 2.3 Phylogenetic analyses of Lineage II and Sisymbrieae

The tribus Sisymbrieae is embedded into the Lineage II of the Brassicaceae family (BrassiBase: Kiefer et al., 2014; Koch et al., 2012, 2018). To test the robustness of the retrieved topologies, we performed phylogenetics on two different taxonomic levels. First dataset (from this point onwards referred to as Lineage II) consisted of newly generated ITS‐based dataset of Sisymbrieae (see first 55 lines in Appendix S1 and Results section) and from BrassiBase retrieved ITS‐based datasets from three other Brassicaceae tribes that together with Sisymbrieae constitute the Lineage II: Brassiceae, Isatideae, and Thelypodieae (BrassiBase: Kiefer et al., 2014; Koch et al., 2012, 2018). The complete list of this dataset with corresponding GenBank entries can be inferred from Appendix S3, datasets A and B. The second dataset consisted of Sisymbrieae accessions only (see below).

Due to the problematic nature of *S. aculeolatum* and *S. afghanicum*, several datasets were investigated within the Lineage II to test for the phylogenetic placement of the two species. Firstly, we tested the position of these two above‐mentioned species by using all four tribes that belong to the Lineage II (sensu BrassiBase). In addition, we tested how presence/absence of *S. aculeolatum* and *S. afghanicum* influence the topology when two outgroups to the Lineage II (Eutremeae and Thlaspideae sensu BrassiBase) are included. The ITS sequences from these two tribes that served as outgroup were retrieved directly from the BrassiBase ITS databank and the complete list of this dataset with corresponding GenBank entries can be inferred from Appendix S3, datasets C and D. Thus, we ended up with four different datasets within Lineage II: Lineage II including *S. aculeolatum* and *S. afghanicum* (Appendix S3, dataset A), Lineage II excluding *S. aculeolatum* and *S. afghanicum* (Appendix S3, dataset B), Lineage II with Eutremeae and Thlaspideae as outgroups including *S. aculeolatum* and *S. afghanicum* (Appendix S3, dataset C), and Lineage II with Eutremeae and Thlaspideae as outgroup excluding *S. aculeolatum* and *S. afghanicum* (Appendix S3, dataset D).

The phylogenetic analyses of Sisymbrieae dataset (consisting of only *Sisymbrium* accessions, see below) as well as the Lineage II dataset (see above) were carried out using Bayesian Inference (BI) showing Bayesian posterior probabilities (BPP) as well as Maximum Likelihood approach (ML), showing likelihood bootstrap support (LBS). BI was carried out using MrBayes v3.2.6 (Ronquist et al., 2012) under GTR + Γ substitution model (alongside the HKY + Γ and SYM + Γ model in Sisymbrieae dataset, see below) using the random‐addition‐sequence method with 10 random perturbations. Two independent Markov chain Monte Carlo (MCMC) analyses of four chains were run—one cold and three heated. Runs were carried out for 20 million cycles, with parameters and trees sampled every 1,000th cycle including an appropriate burn‐in (10%) as inferred from the evaluation of the trace files using Tracer v1.7.0 (Rambaut et al., 2018). Maximum likelihood tree inference relied on RAxML‐HPC v8.2.10 (Stamatakis, 2014) with 1,000 Ml bootstrap iterations with the default number of distinct rate categories. Tree searches were executed from a random maximum‐parsimony tree employing either the GTRCAT or the GTRGAMMA bootstrapping phase model. In case of multi‐locus Sisymbrieae dataset, six partitioning schemes were additionally enforced, one combining the three cpDNA loci and one combining rDNA ITS and ETS. Due to the complex nature of the four analyzed Bra loci (consisting of different number of exons and introns), each of these was assigned its own partition.

After determining whether *S. aculeolatum* and *S. afghanicum* should be incorporated into the dataset based on single‐locus ITS phylogenies, we focussed on the tribe Sisymbrieae. We first generated ITS sequences for 209 accessions representing approximately 85% of all currently recognized *Sisymbrium* species (Appendix S1), using the PCR programme and primers specified in Appendix S2. This helped us to assess the genetic diversity of the genus in general. We then reduced the taxon sample to include only one accession per species (if no divergent ITS sequences were retrieved), or alternatively one accession per diverging ribotype. The reduced taxon sample comprised 55 accessions (first 55 entries in Appendix S1). For these accessions, additional loci were amplified (specified above). All accessions of the following investigated species exhibited identical ambiguous positions in the following loci: *S. loeselii* in ITS and ETS loci, *S. strictissimum* in Bra13, Bra246, Bra813, and Bra1402 loci, *S. subspinescens* in ITS locus and *S. yunnanense* in Bra13 and Bra246 loci. To test for multiple copies within individuals, cloning of the PCR amplicons of one individual per species (depicted in * in the Appendix S1) was carried out using the Thermo Scientific™ CloneJET PCR Cloning Kit according to the instructions of the manufacturer. An exception was *Sisymbrium strictissimum*, in which two accessions (both exhibiting one diverging ITS copy) were investigated for all four aforementioned Bra loci. 10–15 clones per locus per individual were analyzed further. The yielded plasmids were isolated and purified using NucleoSpin® Plasmid EasyPure (Macherey‐Nagel) following the manufacturer's instructions. The sequencing was performed by the Microsynth Seqlab (Gottingen, www.microsynth.ch), using universal pJET1.2_F and pJET1.2_R primers.

While the Lineage II phylogenies were based solely on ITS sequence information, the phylogeny of Sisymbrieae was based on two rDNA loci ITS and ETS, four low copy Bra loci and three cpDNA encoded loci (see first 55 lines in Appendix S1 for taxon sample). Single‐locus phylogenies were carried out, and the topologies were assessed using IQ‐TREE (Nguyen et al., 2015). We concatenated the six nuclear DNA loci and three cpDNA loci, respectively, as no major topological incongruences were observed within the single loci. However, as concatenation might lead to artificial topologies and bias statistical branch support (Kubatko & Degnan, 2007), we additionally tested both, nuclear DNA‐based and cpDNA‐based Sisymbrieae datasets using ASTRAL (Zhang et al., 2017), showing bootstrapping (BS). It was proven that this algorithm is statistically consistent under the multi‐species coalescent model (Mirarab et al., 2014). Both IQ‐TREE analysis as well as ASTRAL analysis were run under default settings, with increased 1,000 bootstrapped gene alignments and 1,000 bootstrapping trees, respectively. The topologies retrieved from Bayesian Inference and Maximum Likelihood were then compared with topologies retrieved from ASTRAL.

### 2.4 Time estimation analyses of Lineage II and Sisymbrieae

To obtain the appropriate crown age of Sisymbrieae, we first dated the whole Lineage II, based on the same ITS‐based taxon set as in the phylogenetic studies, one including the four tribes of the Lineage II only (see Appendix S3, dataset A for the whole accession list) and one including the two additional outgroups (Eutremeae and Thlaspideae sensu BrassiBase, see Appendix S3, dataset C for the whole accession list), both now including *S. aculeolatum* and *S. afghanicum*. These two different datasets were tested to assess the robustness of the retrieved time spans and to allow for additional secondary calibration points in the dataset including Eutremeae and Thlaspideae as outgroups.

There are no reliable Brassicaceae fossils that could potentially serve as time constraints (Franzke et al., 2011), with a possible exception of East European *Bunias* fossil from the Pliocene (Mai, 1995). Furthermore, in the light of general fossil absence, it is hard to establish a stable evolutionary time frame. Thus, we tested three different time constraint approaches, firstly using secondary calibration points retrieved from a whole plastome analysis (Huang et al., 2020) and as an alternative using the internal transcribed spacer substitution rate for herbaceous annual/perennial angiosperms of 4.13 × 10^–9^ sub/site/yr (Kay et al., 2006). The third approach included secondary calibration points as well as the published ITS substitution rate. While Huang et al. (2020) also published the crown age of Sisymbrieae, we did not incorporate this age into our dating analysis of the Lineage II, but used it as an independent checkpoint to estimate the accuracy of the ITS‐based substitution rate calibration as a time constraint. Furthermore, we wanted to test how different datasets might influence the time spans. Our multiple layer approach allowed us to test how time spans might be effected by (a) the taxon sample outside of Sisymbrieae, by using only Lineage II in our study or alternatively using Lineage II with two outgroups sensu BrassiBase versus whole family of Brassicaceae used in Huang et al. (2020); (b) the taxon sample within the tribus of interest, by using a comprehensive taxon sample of Sisymbrieae in our study versus a reduced taxon sample of *Sisymbrium* used in Huang et al. (2020); and (c) calibration strategy, by using single‐gene‐based nuclear substitution rate versus primarily calibrated nodes from a whole plastome phylogeny used in Huang et al. (2020).

After obtaining a robust crown age for the tribus Sisymbrieae from our ITS‐based time divergence estimations of different datasets of Lineage II, we applied the average median time span of all retrieved time spans onto the multi‐locus Sisymbrieae dataset consisting of 55 entries (see first 55 entries in Appendix S1). Due to the topological incongruences between nuclear DNA‐based trees and cpDNA‐based trees (in all ML, BI, and ASTRAL algorithms: see Results), better resolution obtained from the nuclear‐based dataset and ability to use ITS substitution rate as calibration method, we carried out time estimation analysis on nuclear DNA‐based dataset of Sisymbrieae only. Five unlinked datasets were fed to the algorithm, each representing one Bra locus and one representing combined ITS and ETS loci. We left the site and the clock model unlinked and set the trees generated from the independently analyzed loci to be linked.

Molecular dating analyses relied on BEAUTi & BEAST v1.10.4 (Suchard et al., 2018) and the uncorrelated relaxed clock model. The use of the uncorrelated relaxed clock model was justified, after assessing the coefficient of variation in all the trace files, which consistently exceed 0.5 in all of the cases—in the dating analyses of the Lineage II as well as of Sisymbrieae. For both Lineage II datasets (the one with putative outgroups Eutremeae and Thlaspideae and the one without), we used the GTR + Γ substitution model, corresponding tree priors (see below), and three independent Monte Carlo Markov chains (MCMC) runs for 100 million generations, with parameters sampled every 20,000th generation. For the datasets of Sisymbrieae, we used the GTR + Γ and HKY substitution models partition‐wise, corresponding tree priors (see below), and three independent Monte Carlo Markov chains (MCMC) runs for 50 million generations, with parameters sampled every 10,000th generation. Effective sample sizes (ESS) for all estimated parameters were assessed using Tracer v1.6.0 (Rambaut et al., 2018). We excluded the option + I, as it may lead to over parametrization (BEAST manual). TreeAnnotator v1.8.4 (Suchard et al., 2018) was used to discard 10% of the saved trees and annotate the rest of them. Maximum clade credibility tree with median node heights was visualized using FigTree graphical viewer of phylogenetic trees v1.4.3 (http://tree.bio.ed.ac.uk/software/figtree/).

All sequence evolution models used in this study were assessed using the Akaike information criterion (AIC) implemented in the jModelTest2 v2.1.6 (Darriba et al., 2012). All the analyses were carried out at the CIPRES Science Gateway computing facility (Miller et al., 2010). The aligned matrices are available as *.nex files upon request. Recent studies have shown that the impact of the tree priors in Bayesian phylogenetics is in general not as strong as previously thought (Ritchie et al., 2017; Sarver et al., 2019). Nevertheless, one should set the priors accordingly, as they might have an impact on the accuracy of the analysis, especially of those based on mixed inter‐ and intraspecies datasets (Ritchie et al., 2017). Thus, as all of our taxon samples—the Lineage II with and without the putative Eutremeae and Thlaspideae outgroups as well as Sisymbrieae dataset—were not limited to one accession per species only (which would automatically exclude the Coalescence tree prior) and to furthermore allow for potential extinction events (embedded into the Birth‐Death tree prior, but not into Yule tree prior), all analyses were done in parallel using the Yule, Coalescence, and Birth‐Death tree prior.

### 2.5 Ancestral range reconstruction of Sisymbrieae

The time divergence tree from BEAST analysis performed under coalescent tree prior was used for the ancestral range reconstruction. Distribution maps and contemporary range estimations followed the same methodology described in detail in Section 2.1. The analysis was carried out using RASP4 v4.0 ancestral state reconstruction tool (Yu et al., 2015, 2020). DEC, DIVALIKE, and BAYAREALIKE biogeographic models with and without corresponding jumping parameter “+j” were tested using BioGeoBEARS v1.1.1 algorithm implemented in RASP through R (Matzke, 2013a, 2013b, 2014). Following the biogeographic division and evolutionary history of Eurasia and Africa (Hurka et al., 2019; Linder, 2014; Linder et al., 2012; Wesche et al., 2016), eight geographic entities were defined: western Irano‐Turanian floristic region (A), Euro‐Siberian steppe and adjacent semi‐deserts (B), Mediterranean and Northern Africa (C), Mongol‐Chinese steppe, semi‐deserts and deserts (D), European temperate and cool‐mixed forests (E), Caucasus (F), Southern African open grasslands (G), and temperate and subtropical East Asia (H). Due to the geographic distance and evolutionary trajectory, it is highly unlikely that biota of the South African open grasslands migrated from/to areas beside the two most adjacent and climatically similar Mediterranean and/or western Irano‐Turanian floristic region. Thus, only combinations of AG and CG were allowed in ancestral range areas including the South African open grasslands with one other region. The maximum number of areas in which a species can coexist was set to three, following the widely distributed *S. irio* that can be found in maximally three out of eight areas specified above.

## 3 RESULTS

Details on individual alignments of Lineage II and Sisymbrieae can be inferred from Appendix S4. Based on the ITS sequence information, three investigated accessions (see first column in Appendix S1) were placed into morphologically similar genus *Erysimum* and were excluded from further analyses. After assessment of the genetic diversity of *Sisymbrium* based on ITS locus, a reduced representative taxon sample set of Sisymbrieae was used for all the subsequent analyses that covered the whole known genetic diversity within this tribus (see Materials & Methods section, for taxon sample refer to the first 55 lines in Appendix S1). The 10–15 sequenced clones per locus per accession consistently retrieved two major individual genetic copies that were included into all subsequent analyses (see Appendix S1, diverging clone copies depicted in *).

### 3.1 Phylogenetics and divergence time estimation of Lineage II

**FIGURE 2:**
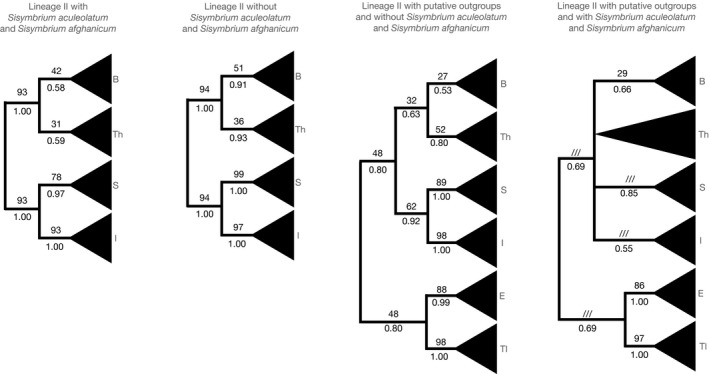
Schematic representation of ITS‐based phylogenetic trees based on different taxon sets, illustrating the topological differences. Values above branches correspond to the LBS (likelihood bootstrap support) values from the maximum likelihood analysis and the values below branches correspond to the BPP (Bayesian posterior probabilities) values of the Bayesian Inference analysis. B, Brassiceae; Th, Thelypodieae; S, Sisymbrieae; I, Isatideae; E, Eutremeae; Tl, Thlaspideae

The intratribal relationships within Lineage II without two outgroups (Eutremeae and Thlaspideae) remained unclear and the monophyly of some tribes remained controversial. Isatideae and Sisymbrieae were retrieved monophyletic (93 LBS/1.00 BPP and 78 LBS/0.93 BPP, respectively), whereas the statistical support for Brassiceae and Thelypodieae was poor—42 LBS/0.58 BPP and 31 LBS/0.59 BPP, respectively (Figure 2). However, in absence of controversial *S. aculeolatum* and *S. afghanicum,* the statistical support for the tribes overall increased to reliable 99 LBS/1.00 BPP for Sisymbrieae and 97 LBS/1.00 BPP for Isatideae and still unreliable 51 LBS/0.91 BPP for Brassiceae and 36 LBS/0.93 BPP for Thelypodieae (Figure 2). The above‐mentioned datasets comprised accessions presented in detail in Appendix S3, datasets A and B. Further statistical features on individual alignments can be inferred from Appendix S4.

The intratribal relationships within Lineage II including two outgroups (Eutremeae and Thlaspideae) remained controversial as well. In dataset without *S. aculeolatum* and *S. afghanicum*, all tribes were retrieved as monophyletic, albeit some with a negligible support. Brassiceae (27 LBS/0.53 BPP) were placed as sister group to Thelypodieae (52 LBS/0.80 BPP), Sisymbrieae (89 LBS/1.00 BPP) were placed as sister clade to Isatideae (98 LBS/1.00 BPP), and Eutremeae (88 LBS/0.99 BPP) were placed as sister group to Thlaspideae (98 LBS/1.00 BPP; Figure 2). When *S. aculeolatum* and *S. afghanicum* were included, the support of previously moderately supported clades plummeted and previously poorly supported topologies collapsed: Eutremeae (86 LBS/1.00 BPP) remained a sister group to Thlaspideae (97 LBS/1.00 BPP) and the true Lineage II was retrieved on a poorly supported comb comprising of Brassiceae (29 LBS/1.00 BPP), Thelypodieae (00 LBS/0.00 BPP), Sisymbrieae (00 LBS/0.85 BPP), and Isatideae (00 LBS/0.55 BPP; Figure 2). The above‐mentioned datasets comprised accessions presented in detail in Appendix S3, datasets C and D. Further statistical features on individual alignments can be inferred from Appendix S4.

The Maximum Likelihood analysis and Bayesian Inference analysis of Lineage II consistently placed both *S. altissimum* and *S. septulatum* as the sister group to the rest of the Sisymbrieae tribe, albeit with a low statistical support (cannot be inferred from collapsed topologies in Figure 2). Alternatively, depending on the tree prior in the time divergence estimation, an alternative sister group to the rest of Sisymbrieae was recognized, consisting of *S. aculeolatum*, *S. afghanicum*, *S. altissimum,* and *S. septulatum*. The overall topology corresponded with the previous study of *Sisymbrium* based on an extensive taxon dataset from Warwick et al., 2002. The used taxon sample of Lineage II allowed for three (or five, when the putative outgroups Eutremea and Thlaspideae were included) secondary calibration points from a fossil‐calibrated whole plastome analysis (Huang et al., 2020). The crown age of Sisymbrieae was not incorporated into the secondary calibrated Lineage II analysis but was used as an independent checkpoint to estimate the accuracy of the use of ITS‐based substitution rates as a time constraint and to test the influence of completeness of a taxon sample. The ESS values of all the time estimation analyses exceeded 400. Secondarily calibrated analyses and analyses calibrated with substitution rates showed highly congruent results in terms of topology, but in some cases only partially congruent time spans (Tables 1 and 2). Nevertheless, the crown age of Sisymbrieae was consistently placed in the Miocene. The combinatorial calibration approach in which both secondary calibration points as well as ITS‐based substitution rate was used placed the mean crown age of Sisymbrieae at the end of early Miocene and the beginning of middle Miocene in corresponding priors and datasets, respectively (Tables 1 and 2). These age spans are highly congruent with the Sisymbrieae crown age from Huang et al., 2020 estimated to be 10.2–18.7 MYA 95% HDP. Similar time spans were inferred from calibration using ITS substitution rate only, consistently placing the mean crown age of Sisymbrieae into the early Miocene. Datasets where only secondary calibration points from Huang et al., 2020 were used, estimated the mean age of Sisymbrieae slightly younger, placing it in the late Miocene (Tables 1 and 2).

**TABLE 1:** Statistics of the crown ages of tribes depending on the calibration method based on the dataset of Lineage II

Calibration method	Tree Prior	Crown age of Brassiceae	Crown age of Isatideae	Crown age of Sisymbrieae	Crown age of Thelypodieae
Secondary calibration (Huang et al., 2020)	BiDe	**Fixed at** 14.3–19.8 MYA 95% HPD	**Fixed at** 5.3–11.2 MYA 95% HPD	**6.87–10.57–13.03 MYA 95% HPD**	**Fixed at** 8.8–17.5 MYA 95% HPD
Secondary calibration (Huang et al., 2020) + ITS substitution rate (Kay et al., 2006)	BiDe	**Fixed at** 14.3–19.8 MYA 95% HPD	**Fixed at** 5.3–11.2 MYA 95% HPD	**10.93–16.30–18.54 MYA 95% HPD**	**Fixed at** 8.8–17.5 MYA 95% HPD
ITS substitution rate (Kay et al., 2006)	BiDe	**27.23–32.94–38.99 MYA 95% HPD**	**7.95–11.20–15.07 MYA 95% HPD**	**12.46–16.62–21.08 MYA 95% HPD**	**10.07–18.48–28.25 MYA 95% HPD**
Secondary calibration (Huang et al., 2020)	Coal	**Fixed at** 14.3–19.8 MYA 95% HPD	**Fixed at** 5.3–11.2 MYA 95% HPD	**5.68–9.52–12.86 MYA 95% HPD**	**Fixed at** 8.8–17.5 MYA 95% HPD
Secondary calibration (Huang et al., 2020) + ITS substitution rate (Kay et al., 2006)	Coal	**Fixed at** 14.3–19.8 MYA 95% HPD	**Fixed at** 5.3–11.2 MYA 95% HPD	**11.50–17.66–25.15 MYA 95% HPD**	**Fixed at** 8.8–17.5 MYA 95% HPD
ITS substitution rate (Kay et al., 2006)	Coal	**37.69–48.36–61.05 MYA 95% HPD**	**7.53–12.55–17.35 MYA 95% HPD**	**13.91–21.15–28.60 MYA 95% HPD**	**9.48–28.70–48.00 MYA 95% HPD**
Secondary calibration (Huang et al., 2020)	Yule	**Fixed at** 14.3–19.8 MYA 95% HPD	**Fixed at** 5.3–11.2 MYA 95% HPD	**7.39–10.61–13.1 MYA 95% HPD**	**Fixed at** 8.8–17.5 MYA 95% HPD
Secondary calibration (Huang et al., 2020) + ITS substitution rate (Kay et al., 2006)	Yule	**Fixed at** 14.3–19.8 MYA 95% HPD	**Fixed at** 5.3–11.2 MYA 95% HPD	**11.39–15.96–18.87 MYA 95% HPD**	**Fixed at** 8.8–17.5 MYA 95% HPD
ITS substitution rate (Kay et al., 2006)	Yule	**26.4–33.01–36.86 MYA 95% HPD**	**7.95–12.66–15.3 MYA 95% HPD**	**12.64–17.92–20.90 MYA 95% HPD**	**9.65–18.52–26.51 MYA 95% HPD**

Note

Time span is indicated by the lower boundary, median age, and the upper boundary. Yule, Yule Tree Prior; Coal, Coalescent Tree Prior; BiDe, Birth‐Death Tree Prior.

Bold values are statistics of the crown ages of tribes depending on the calibration method based on the dataset of Lineage II.

**TABLE 2:** Statistics of the crown ages of tribes depending on the calibration method based on the dataset of Lineage II with two putative outgroups

Calibration method	Tree Prior	Crown age of Brassiceae	Crown age of Isatideae	Crown age of Sisybrieae	Crown age of Thelypodieae	Crown age of Eutremeae	Crown age of Thlaspideae
Secondary calibration (Huang et al., 2020)	BiDe	**Fixed at** 14.3–19.8 MYA 95% HPD	**Fixed at** 5.3–11.2 MYA 95% HPD	**8.09–11.62–14.00 MYA 95% HPD**	**Fixed at** 8.8–17.5 MYA 95% HPD	**Fixed at** 6.9–15.1 MYA 95% HPD	**Fixed at** 9.5–17.1 MYA 95% HPD
Secondary calibration (Huang et al., 2020) + ITS substitution rate (Kay et al., 2006)	BiDe	**Fixed at** 14.3–19.8 MYA 95% HPD	**Fixed at** 5.3–11.2 MYA 95% HPD	**11.59–15.92–19.39 MYA 95% HPD**	**Fixed at** 8.8–17.5 MYA 95% HPD	**Fixed at** 6.9–15.1 MYA 95% HPD	**Fixed at** 9.5–17.1 MYA 95% HPD
ITS substitution rate (Kay et al., 2006)	BiDe	**26.09–32.16–35.90 MYA 95% HPD**	**7.77–13.04–16.37 MYA 95% HPD**	**13.14–18.70–22.34 MYA 95% HPD**	**9.33–18.27–29.94 MYA 95% HPD**	**11.44–16.95–20.7** **MYA 95% HPD**	**13.35–18.48–21.52 MYA 95% HPD**
Secondary calibration (Huang et al., 2020)	Coal	**Fixed at** 14.3–19.8 MYA 95% HPD	**Fixed at** 5.3–11.2 MYA 95% HPD	**6.8–10.95–14.49 MYA 95% HPD**	**Fixed at** 8.8–17.5 MYA 95% HPD	**Fixed at** 6.9–15.1 MYA 95% HPD	**Fixed at** 9.5–17.1 MYA 95% HPD
Secondary calibration (Huang et al., 2020) + ITS substitution rate (Kay et al., 2006)	Coal	**Fixed at** 14.3–19.8 MYA 95% HPD	**Fixed at** 5.3–11.2 MYA 95% HPD	**12.23–19.56–24.81 MYA 95% HPD**	**Fixed at** 8.8–17.5 MYA 95% HPD	**Fixed at** 6.9–15.1 MYA 95% HPD	**Fixed at** 9.5–17.1 MYA 95% HPD
ITS substitution rate (Kay et al., 2006)	Coal	**35.23–44.74–53.28 MYA 95% HPD**	**7.77–13.58–18.77 MYA 95% HPD**	**14.29–22.2–29.90 MYA 95% HPD**	**11.53–38.52–49.07 MYA 95% HPD**	**11.77–19.75–27.07 MYA 95% HPD**	**16.41–23.27–28.9** **MYA 95% HPD**
Secondary calibration (Huang et al., 2020)	Yule	**Fixed at** 14.3–19.8 MYA 95% HPD	**Fixed at** 5.3–11.2 MYA 95% HPD	**8.14–11.85–19.00 MYA 95% HPD**	**Fixed at** 8.8–17.5 MYA 95% HPD	**Fixed at** 6.9–15.1 MYA 95% HPD	**Fixed at** 9.5–17.1 MYA 95% HPD
Secondary calibration (Huang et al., 2020) + ITS substitution rate (Kay et al., 2006)	Yule	**Fixed at** 14.3–19.8 MYA 95% HPD	**Fixed at** 5.3–11.2 MYA 95% HPD	**11.76–15.70–19.61 MYA 95% HPD**	**Fixed at** 8.8–17.5 MYA 95% HPD	**Fixed at** 6.9–15.1 MYA 95% HPD	**Fixed at** 9.5–17.1 MYA 95% HPD
ITS substitution rate (Kay et al., 2006)	Yule	**25.23–30.74–34.95 MYA 95% HPD**	**8.07–12.32–15.99 MYA 95% HPD**	**12.71–17.81–21.68 MYA 95% HPD**	**8.93–16.50–28.26 MYA 95% HPD**	**11.15–16.16–20.25 MYA 95% HPD**	**13.35–17.66–21.04 MYA 95% HPD**

Note

Time span is indicated by the lower boundary, median age and the upper boundary. Yule, Yule Tree Prior; Coal, Coalescent Tree Prior; BiDe, Birth‐Death Tree Prior.

Bold values are statistics of the crown ages of tribes depending on the calibration method based on the dataset of Lineage II with two putative outgroups.

### 3.2 Phylogenetics and divergence time estimation of Sisymbrieae

**FIGURE 3:**
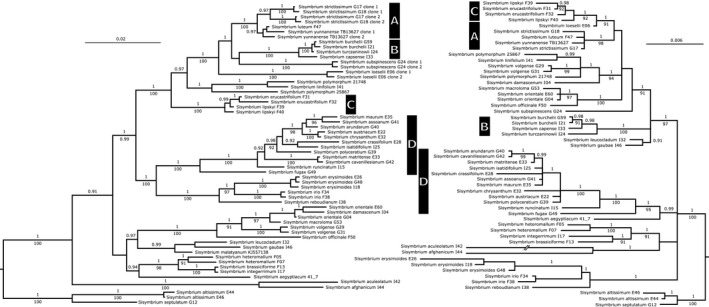
Bayesian Inference phylogenetic tree of Sisymbrieae, the nuclear DNA tree (left) and the cpDNA tree (right). Values below branches correspond to the LBS (likelihood bootstrap support) values from the maximum likelihood analysis (values < 0.90 are not shown) and the values above branches correspond to the BPP (Bayesian posterior probabilities) values of the Bayesian Inference analysis (values < 0.90 are not shown). a, disjunct forest Eurasian clade; b, South African clade; c, Caucasian clade; d, pure Mediterranean clade. Two slashes indicate a branch that has been artificially shortened. Codes next to species names refer to the isolation codes from Appendix S1

The separately concatenated nuclear DNA loci and cpDNA loci, respectively, showed only partially congruent results. The most prominent difference was a strong bias in the resolution (Figure 3). While the nuclear DNA loci have contributed substantially to a well‐resolved backbone of the Sisymbrieae phylogeny but in some cases failed to unravel internal topology, the three analyzed cpDNA loci recovered poorly resolved backbone, but in contrast allowed for better‐resolved internal nodes. The ASTRAL analyses (Appendices S5 and S6) retrieved trees with topologies highly comparable to the ones resulting from concatenated datasets (Figure 3).

Similarly to the phylogenetic reconstructions and time estimation analyses of the Lineage II datasets, the time estimation analysis of the Sisymbrieae dataset did not consistently recognize the same Sisymbrieae lineages as sister clade to the rest of the tribe (topologies not shown). Again, depending on the tree prior, a poorly supported group consisting of either *S. aculeolatum*, *S. afghanicum*, *S. altissimum,* and *S. septulatum* or of only *S. aculeolatum* and *S. afghanicum* was placed as the sister clade toward the rest of Sisymbrieae. We reconstructed the outgroup following the topology of some of our time estimation analyses and previous phylogenies with an extensive taxon sample (Warwick et al., 2002), where *Sisymbrium altissimum* and *S. septulatum* constituted a sister clade to the rest of Sisymbrieae (Figure 3, and Appendices S5 and S6). Independently, the phylogenies uncovered numerous geographically well‐defined clades that remained well‐supported in nuclear DNA based as well as cpDNA‐based analyses, regardless of the used dataset (nuclear DNA and cpDNA) as well as algorithms (ML, BI, ASTRAL: Figure 3, and Appendices S5 and S6).

These included central Asian *Sisymbrium brassiciforme*, *S. heteromallum,* and Iranian endemic *S. integerrimum* (0.98 LBS/1.00 BPP/99 BS NT; 0.92 LBS/1.00 BPP/00 BS CP), Mediterranean and Middle Eastern *S. erysimioides*, *S. irio,* and *S. reboudianum* (100 LBS/1.00 BPP/100 BS NT; 100 LBS/1.00 BPP/00 BS CP), pure (primarily West) Mediterranean *S. austriacum*, *S*. *assoanum*, *S. arundanum*, *S. cavanillesianum*, *S. chrysanthum*, *S. crassifolium*, *S. fugax*, *S. isatidifolium*, *S. matritense*, *S. maurum*, *S. polyceratium,* and *S. runcinatum* (100 LBS/1.00 BPP/96 BS NT; 0.95 LBS/1.00 BPP/94 BS CP), South African *S. capense*, *S. burchelii,* and *S. turczaninowii* (100 LBS/1.00 BPP/100 BS NT; 100 LBS/1.00 BPP/100 BS CP), Caucasian *S. erucastrifolium* and *S. lipskyi* (100 LBS/1.00 BPP/100 BS NT; 0.91 LBS/1.00 BPP/90 BS CP) and disjunct forest Eurasian *S. luteum*, *S. strictissimum,* and *S. yunnanense* (100 LBS/1.00 BPP/100 BS NT; 0.98 LBS/1.00 BPP/97 BS CP). Three major incongruences between the nucleotide and plastid phylogenetic signal were spotted. While the *Sisymbrium polymorphum* s.l. (consisting of *S. polymorphum* and *S. linifolium*) represented a fairly isolated lineage in the nuclear‐based phylogenies, it was placed in chloroplast phylogenies as sister group to *S. volgense* (0.89 LBS/1.00 BPP/00 BS). Another contradicting position concerned *Sisymbrium damascenum*, which was nested within the *Sisymbrium macroloma*—*S. officinale*—*S. orientale*—*S. volgense* clade in the nuclear tree, while in the chloroplast tree, it clustered together with *S. polymorphum* s.l. and *S. volgense*. An additional minor topological change was observed in fairly isolated species in nuclear‐based phylogenies *S. loeselii* that was placed as sister group to the Caucasian clade in chloroplast‐based phylogenies (0.90 LBS/1.00 BPP/86 BS).

**FIGURE 4:**
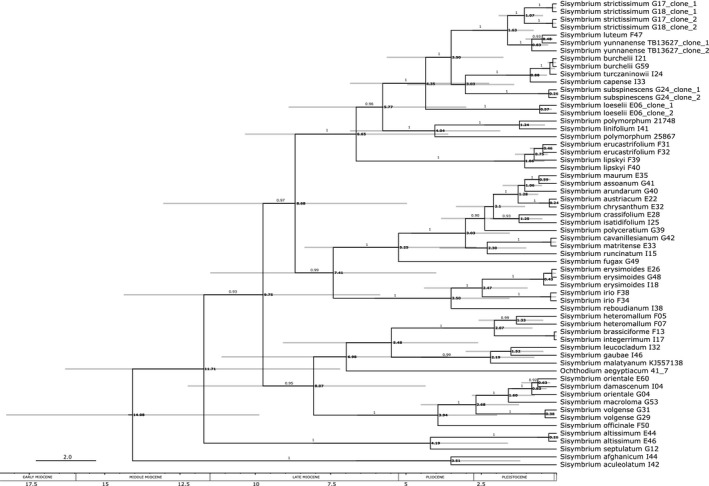
Dated nuclear DNA phylogenetic tree of Sisymbrieae retrieved from BEAST analysis under Coalescent tree prior. The intervals next to the nodes represent the time span of the node age, including the 95% HDP. Absolute ages are in millions of years. The numbers on the branches are statistical support values (Bayesian posterior probabilities, values < 0.90 are not shown). The bold numbers next to nodes represent median ages in millions of years. Codes next to species names refer to the isolation codes from Appendix S1

The position of *O. aegyptiacum* remained controversial, albeit the whole Lineage II phylogeny doubtlessly placed this taxon in *Sisymbrium*. All species with multiple accessions, except *Sisymbrium polymorphum*, were resolved as monophyletic with maximal support or in the cases of *S. orientale* and *S. yunnanense* paraphyletic with low statistical support. Notably, *S. brassiciforme* and *S. integerrimum* exhibited almost identical nucleotide sequences, yet showed extensive differences in the investigated chloroplast regions. Nevertheless, these species showed a sister group relationship in nucleotide as well as chloroplast set (Figure 3, Appendices S5 and S6). The ESS values of time estimation analyses exceeded 600 and the different tree priors did not majorly influence the time spans (see Table 3). Four geographically well‐defined clades were retrieved from the analyses. The mean stem age of the disjunct forest Eurasian clade was dated to the Pliocene/Pleistocene, while its mean crown age was dated to early Pleistocene. The mean stem age of the South African clade was dated to the Pliocene, while its mean crown age was dated to the Calabrian in the Pleistocene (Figure 4). Furthermore, the mean stem age of the Caucasian clade was dated earlier to the late Miocene, while its mean crown age was dated as well to the Calabrian in the Pleistocene, and the mean stem as well as crown age of the Mediterranean clade was dated to the late Miocene (Figure 4). Retrieved clone copies from *S. strictissimum* (100 LBS/1.00 BPP), *S. subspinescens* (100 LBS/1.00 BPP), and *S. loeselii* (100 LBS/1.00 BPP) formed well‐supported sister group relationships (Figure 3 and Figure 4). Poorly supported paraphyly between *Sisymbrium yunnanense* clones and its sister species *S. luteum* (48 LBS/0.92 BPP) was observed (Figure 3, Figure 4).

**TABLE 3:** Statistics of the crown and stem ages of geographically defined clades depending on the calibration method based on the dataset of Sisymbrieae

Geographic entity	Age	Coal	Yule	BiDe
Disjunct forest Eurasian clade (A)	Stem	1.76–3.50–5.64 MYA 95% HPD	2.44–4.40–6.73 MYA 95% HPD	2.23–4.20–6.56 MYA 95% HPD
	Crown	0.76–1.63–2.79 MYA 95% HPD	1.10–2.19–3.60 MYA 95% HPD	0.97–2.03–3.43 MYA 95% HPD
South‐African clade (B)	Stem	1.41–3.03–4.96 MYA 95% HPD	2.11–3.85–6.03 MYA 95% HPD	1.92–3.66–5.89 MYA 95% HPD
	Crown	0.32–0.88–1.69 MYA 95% HPD	0.47–1.19–2.22 MYA 95% HPD	0.43–1.10–2.11 MYA 95% HPD
Caucasian clade (C)	Stem	3.6–6.65–10.34 MYA 95% HPD	4.69–8.06–12.18 MYA 95% HPD	4.29–7.72–11.81 MYA 95% HPD
	Crown	0.4–1.06–2.08 MYA 95% HPD	0.62–1.43–2.7 MYA 95% HPD	0.54–1.33–2.51 MYA 95% HPD
Mediterranean clade (D)	Stem	4.97–8.68–13.06 MYA 95% HPD	6.1–10.07–14.76 MYA 95% HPD	5.68–9.67–14.44 MYA 95% HPD
	Crown	4.00–7.41–11.5 MYA 95% HPD	5.1–8.86–13.36 MYA 95% HPD	4.72–8.48–13.18 MYA 95% HPD

Note

Time span is indicated by the lower boundary, median age and the upper boundary. Yule, Yule Tree Prior; Coal, Coalescent Tree Prior; BiDe, Birth‐Death Tree Prior.

### 3.3 Ancestral range reconstruction of Sisymbrieae

**FIGURE 5:**
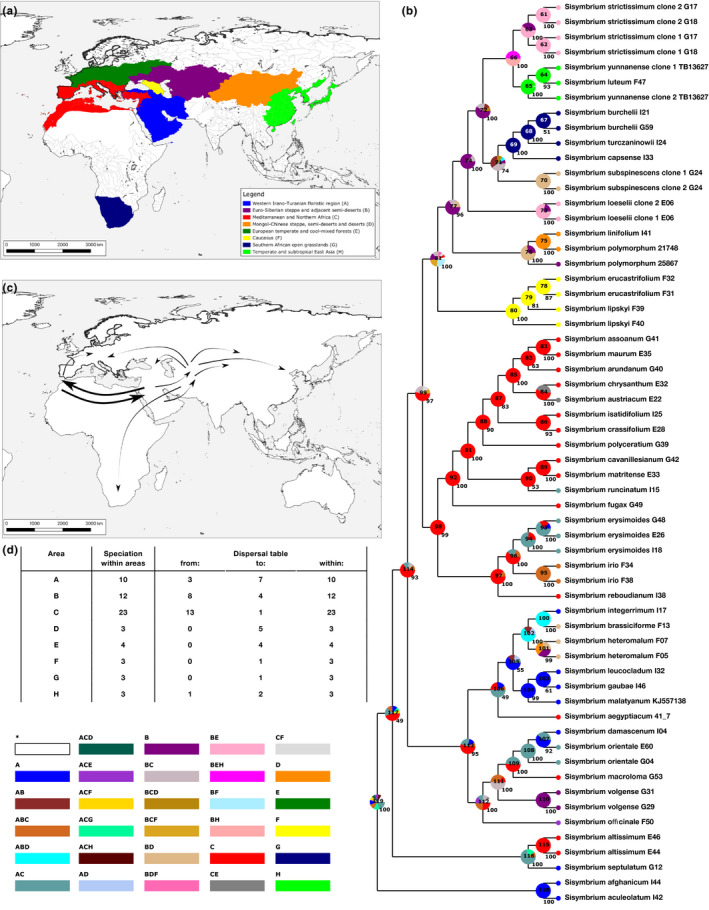
Ancestral range reconstruction, based on a dated nuclear DNA phylogenetic tree of Sisymbrieae retrieved from BEAST analysis under Coalescent tree prior. (a) Geographic representation of biogeographic entities, (b) tree representation of ancestral range reconstruction based on a time divergence tree retrieved from BEAST analysis under Coalescent tree prior, numbers in the middle of pie charts indicate node identity and correspond to the node identity in Appendix S7. Numbers next to pie charts correspond to node frequencies and codes next to species names refer to the isolation codes from Appendix 1, (c) visual representation of the most common range changes, where the arrowheads indicate the direction of the distribution shift/expansion and thickness of the arrows the frequency, (d) speciation and dispersal per biogeographic entity defined in (a). Legend in the bottom left corner correspond to the colors in (a) and (b)

The most optimal model according to the AIC algorithm was DEC + j (Appendix S7). Ancestral range reconstruction placed the origin of the tribus Sisymbrieae into the western Irano‐Turanian floristic region and Mediterranean (Figure 5a and 5b). In the latter, the highest number of speciation events i. e. 23 was inferred, followed by Euro‐Siberian steppe and adjacent semideserts with 12 and western Irano‐Turanian floristic region with 10 speciation events (Figure 5d). These three areas were also the major source for dispersal events, 13 of which took place from the Mediterranean region (Figure 5d). Dispersal between areas index also pointed toward a close relationship between Mediterranean and western Irano‐Turanian floristic region, retrieving eight such dispersal events, albeit with a strong bias, as seven out of eight dispersal events had their onset in the Mediterranean and dispersed into the western Irano‐Turanian floristic region (Figure 5b and 5c). Most of the dispersal routes followed from Mediterranean and/or through western Irano‐Turanian floristic region (Figure 5c) from which the routes separated into western ones that invaded Europe through the Eastern European Plain (Figure 5b nodes 81 → 80 and 74 → 73) and eastern ones that invaded eastern portion of continental Asia (Figure 5b nodes 77 → 76 and 113 → 105→100). The node transition 72 → 66 illustrates an expansion to the east into temperate and subtropical East Asia as well as to the west into European temperate and cool‐mixed forests from central laying contemporary Euro‐Siberian steppes and adjacent semideserts that were at that point also covered in forests (see Discussion). Europe was also invaded through Mediterranean (Figure 5b nodes 85 → 84). The Southern African grasslands were invaded from climatically similar Mediterranean and adjacent dry habitats of Middle Asia (Figure 5b nodes 71 → 69). The corresponding statistics from the ancestral range reconstruction can be further inferred from Appendix S7. Please note that *S. linifolium* is the only *Sisymbrium* species whose distribution extends into the North American prairies, however, for easier depiction North America is not illustrated on the Figure 5.

## 4 DISCUSSION

While the onset and development of flora of the Mediterranean is relatively well investigated (Barres et al., 2013; Guzmán & Vargas, 2005; Manafzadeh et al., 2014; Molins et al., 2011; Ortiz et al., 2008; Scheunert & Heubl, 2014; Tremetsberger et al., 2016), the florogenesis of the Eurasian steppe and Middle East is rather poorly understood. This study focuses on *Sisymbrium* L.—yellow‐flowering herbaceous Brassicaceae, which grows predominantly in dry steppe, semidesert, and rural habitats of Northern hemisphere (Figure 1).

### 4.1 Taxon sampling strategy and differences in the topology

Following the contemporary big scale phylogenies, genetic as well as cytological evidence, we excluded taxa *Orychophragmus* (Al‐Shehbaz, 1985; Couvreur et al., 2010; German et al., 2009; Gomez‐Campo, 1980; Liu et al., 2012; Lysak et al., 2007) and *Sinalliaria* (Kiefer et al., 2014; Koch et al., 2012, 2018) from the analysis, whereas *Sisymbrium leucocladum* (Boiss.) D.A.German & Al‐Shehbaz (=*Pseudofortunya leucoclada* (Boiss.) Khosravi) and *O. aegyptiacum* remain included (Nikolov et al., 2019; Walden et al., 2020). Our Lineage II phylogenetics and the high morphological resemblance of *S. aculeolatum* and *S. afghanicum* to other *Sisymbrium* species (with a notable exception of purple flowers versus yellow flowers in all other *Sisymbrium* species but *S. leucocladum*) indicate that the two aforementioned species belong to the genus *Sisymbrium*. The strong impact these two species have on tree topologies might be an indication of an ancient hybrid origin or intense introgression—both known to disrupt the phylogenetic structure of bifurcating trees. This would also explain why *S. aculeolatum* was not retrieved as a part of Sisymbrieae in Walden et al. (2020). Further analyses, however, are necessary for any robust conclusions on this issue. The overall topology of all Lineage II phylogenetic trees (ML and BI‐based) was in correspondence with other studies that have analyzed phylogenetic aspects of Brassicaceae evolution and included *Sisymbrium* species (Huang et al., 2020; Nikolov et al., 2019; Warwick et al., 2002). Due to the topological incongruences, the nuclear‐ and chloroplast‐based matrices in Sisymbrieae dataset were not concatenated and are presented as separate phylogenetic reconstructions in Figure 3 (ML and BI analyses) and Appendices S5 and S6 (ASTRAL analyses).

While the clades A (European *S. strictissimum* and East Asian *S. luteum* and *S. yunnanense*), B (South African clade), and C (Caucasian clade) were recognized already by Warwick et al., 2002, an additional geographically well‐defined Mediterranean clade D was retrieved in this study (Figures 3 and 4, Appendices S5 and S6). The minor topological change in the placement of *S. loeselii* and closer relationship to *S. erucastrifolium* in the chloroplast‐based phylogenies fits well with the morphological characteristics shared between these two species. Contrarily, major topological difference in the placement of *Sisymbrium volgense* might be attributed to its hybrid nature, where the progenitor not only genetically (this study) but also morphologically highly resembles the putative maternal lineage *Sisymbrium polymorphum* s.l. (Dorofeyev, 1997). Surprisingly, according to the nuclear signals, genetically distantly related *Sisymbrium damascenum* is placed in close proximity to the *S. polymorphum* s.l. and *S. volgense* in the chloroplast‐based tree. This relationship cannot be explained neither by morphological similarities nor by geographical proximity and further genome‐wide research would be necessary to irrefutably elucidate this peculiar topological incongruence.

The initial study of Mutlu and Karakuş (2015) associated *Sisymbrium malatyanum* endemic to Turkey with central Asian *S. brassiciforme* and *S. heteromallum*, albeit with poor statistical support. This placement could not be explained well either by morphology or by biogeography. Contrarily, a more comprehensive taxon sampling of this study placed it into a clade with *S* *isymbrium gaubae* and *S. leucocladum* with a high statistical support (0.75 LBS/0.99 BPP/90 BS) (Figure 3). Both of these species are endemic to Iran, and *S. gaubae* habitually resembles *S. malatyanum*, further supporting this phylogenetic placement. As for the pair *Sisymbrium integerrimum* and *S. brassiciforme,* as early as 1968 Hedge already speculated that the two are very closely related species, where the former might represent a marginal form of the latter. Five shared identical nuclear‐based loci support the formal synonymization of these two names (Khodashenas & Assadi, 2007).

### 4.2 Differences in the dating and retrieval of multiple copies

There have been some substantial differences in the employed calibration methods, datasets, and matrices. Huang et al. (2020) used a generally slowly evolving whole plastome datasets of a representative taxon sample of rosids, where reliable fossils are available, to constrain early nodes. Subsequently, they applied these now secondary constraints to the ITS‐based tribe levels under the assumption that plastome and genome‐based time estimations produce converging results (Hohmann et al., 2015; Huang et al., 2016). Contrarily, we applied the average ITS‐based substitution rate for herbaceous annual/perennial plant species on a reduced taxon sample, covering not all Brassicaceae tribes, but only Lineage II with and without the two putative outgroups (Eutremeae and Thlaspideae). There were some differences in the time spans of crown ages of Brassiceae, Isatideae, and Thelypodieae between Huang et al. (2020) and our analysis (Tables 1 and 2). These time frame shifts can be easily explained by different time constraint strategies with different backgrounds as well as by differences in the taxon sets. Interestingly, when only secondary calibration points from Huang et al. (2020) are used in our study, the crown age of Sisymbrieae does not correspond with the crown age inferred from Huang et al. (2020). This illustrates how much of an influence different taxon samples has on the retrieved time spans. Nevertheless, the crown age of Sisymbrieae remained relatively stable in the present study, regardless of the taxon sample and used constraints, indicating that at least within Sisymbrieae, secondary calibration based on plastome datasets indeed recovers highly congruent results with the ITS‐based substitution rate calibration (Huang et al., 2020).

The whole Brassicaceae family is characterized by an α‐WGD, which is only a recent one in the series of two additional older known WGDs that predated the split of Brassicaceae. These were followed by an extensive diploidization and shaped the evolutionary history of whole angiosperms and Brassicales, respectively (Barker et al., 2009; Fawcett et al., 2009; Franzke et al., 2011; Huang et al., 2020; Mandáková et al., 2017; Schranz et al., 2012). Recent studies have shown that approximately 40% of all Brassicaceae underwent an additional neopolyploidization event that has not yet been followed by diploidization (Hohmann et al., 2015; Kagale et al., 2014). Despite the fact that there is no evidence that *Sisymbrium* underwent a recent polyploidization event, several nuclear‐encoded multi‐copy loci (marked with * in the Appendix S1) in four species possessed two different copies, however, in all of the cases the copies clustered monophyletically. This indicates that the copies are probably remnants of polyploidization events and are not of hybrid nature. While there were always only two copies that were retrieved from the majority of the clones, individual SNPs, and chimeric copies were also detected (however, only in 1–2 clones out of 15 analyzed). These can be attributed to sequencing errors, paralogous nature of loci (as none of the investigated nuclear DNA loci were single copy), or cloning artifacts.

### 4.3 Biogeography of *Sisymbrium* in South Africa

We hypothesize that *Sisymbrium* migrated to southern Africa from the Irano‐Turanian floristic region through the East African Riff Mountains sometime in the Pliocene (stem ages dated to approximately 3.5–4.0 MYA: Figure 4 and Figure 5). During that time, this region was under strong influence of Pliocene uplift, which arguably had the most prominent effect on its present day topography (Partridge & Maud, 1987). During the Late Pliocene, an asymmetrical uplift of the whole continent and the consequent formation of the Post‐African II erosion surface took place (Partridge & Maud, 1987). These resulted in a development of a temperature as well as precipitation gradient. Middle and Late Pleistocene climate fluctuations have caused, together with the eustatic sea‐level changes, a reduction and shift in the precipitation profile. During glacial cycles, the regions that nowadays experience summer rainfall were under winter rainfall regime (Hoag & Svenning, 2017; van Zinderen Bakker, 1978). Cold air masses that were produced more often due to the development of the Antarctic polar cap were penetrating deeply into the African subcontinent (van Zinderen Bakker, 1978). During glacial periods, open dry grassland and semidesert environments were favored (Knight & Grab, 2016; Partridge & Maud, 1987) in which dry‐adapted *Sisymbrium* species could thrive. This is also supported by our time estimation analysis, which placed the crown age of South African *Sisymbrium* species into the same period (Figure 4). Plant disjunctions between the Mediterranean, Irano‐Turanian floristic region, and southern Africa are a common phenomenon tightly connected to the xeric conditions that connect all three areas (Goldblatt, 1978). While the most common explanation for such a disjunction is the spread from southern Africa northwards (Désamoré et al., 2011; Durvasula et al., 2017; Klak et al., 2017; Moore et al., 2010; Valente et al., 2011), our dataset indicates that *Sisymbrium* occupied southern Africa from the Irano‐Turanian floristic region, a more seldom pattern observed in only some other plant species (Carlson et al., 2012; Pyankov et al., 2002).

### 4.4 Biogeography of *Sisymbrium* in the Mediterranean

The Mediterranean climate regime we know today was established around 3.2 MYA (Suc, 1984). Changes in the precipitation reduction and development of seasonality resulted in a compositional and structural change of the Mediterranean forests that lost subtropical elements and started to resemble contemporary Mediterranean forests with drought‐adapted species (Kondraskov et al., 2015; Thompson, 2005). Similarly to the Eurasian steppe belt, the Mediterranean basin was also under strong influence of Pleistocene climatic oscillations causing recurrent range shifts that have shaped current species’ distributions (Hewitt, 2011; Weiss & Ferrand, 2007). Prior to the *Sisymbrium* migration into the Mediterranean, this basin was already under strong influence of gradual global cooling and aridification, which have been initiated as early as in the middle Miocene (van Dam, 2006; Zachos et al., 2008).

The Mediterranean migration of *Sisymbrium* from the western Irano‐Turanian floristic region coincides with the Messinian Salinity Crisis, characterized by massive desiccation of the Mediterranean Sea, which started in the middle Miocene (early Serravallian, 13.0 MYA) and ended with the Zanclean Reflooding around 5.33 MYA (Fauquette et al., 2006; Krijgsman et al., 2010). Extensive pollen record dated to the same period also indicates that Anatolian steppes dominated by *Artemisia* and *Ephedra* spread into the Mediterranean from the western Irano‐Turanian floristic region. The earliest documentations of open dry habitats in the southern Mediterranean are dated much later to roughly 3.5 MYA, when the mangrove forests dried up and gave space to open steppe vegetation. Approximately 2.5 MYA, the mesophilous forests of the northern Mediterranean drew northwards making room for more open environments known as *Artemisia* steppes (Suc et al., 2018). The peak of the Messinian salinity crisis followed by stepwise expansion of drier open habitats coincides with the deeper nodes indicating diversification of Mediterranean *Sisymbrium* species, which started around the transition to the Pleistocene. The onset of modern open grasslands on the Iberian Peninsula is dated to around 1.0 MYA (González‐Sampériz et al., 2010). With its complex topography and relatively stable climate during the Late Quaternary, the Iberian Peninsula served as a Pleistocene refugial center for numerous plant species (Dubreuil et al., 2008; Heredia et al., 2007; Médail & Diadema, 2009; Pérez‐Collazos et al., 2009) and could have potentially endorsed diversification of *Sisymbrium* species. High number of *Sisymbrium* species (up to 12, depending on the taxonomic treatments) on the Iberian Peninsula (Ball, 1964; Pujadas Salvá, 1993) also points toward this region being a shelter during the unfavorable conditions (Figures 4 and 5). High number of speciation and dispersal events within and from this region furthermore reflects an extremely dynamic nature of the Mediterranean (Figure 5b and d). A clade consisting of Mediterranean *Sisymbrium damascenum*, *S. orientale,* and *S. macroloma* points toward another independent invasion of the Mediterranean that took place after the end of the Messinian salinity crisis and continued throughout the Pleistocene. Contemporary distribution patterns of these species again indicate that the invasion probably started from the western Irano‐Turanian region (Figures 4 and 5).

### 4.5 Biogeography of *Sisymbrium* in the Irano‐Turanian region & Caucasus

The western part of the Irano‐Turanian floristic region is one of the global biodiversity hotspots of the Old World (Mittermeier et al., 2000) and a putative origin of several important crop wild relatives, including the family of Brassicaceae (Franzke et al., 2011; Hedge, 1976; Karl & Koch, 2013; Noroozi et al., 2019). Furthermore, due to its position connecting the eastern and western Eurasian floras, this region has also been proposed to be the main source of plant taxa for adjacent floristic regions, especially the Mediterranean (Herrera, 2010; Jabbour & Renner, 2012; Manafzadeh et al., 2014; Quezel, 1985; Thompson, 2005, this study).

This region has experienced a predominantly dry and stable climate since the early Eocene (Manafzadeh et al., 2017). Mountain ranges of Alborz, Zagros, Kopet Dagh, and Pamir had formed from the middle Miocene through the early Pliocene creating considerable habitat heterogeneity (Bobek, 1937, 1953; Manafzadeh et al., 2017). Furthermore, climate stability of this region might also sustain genetic diversity, as it deters from climate‐dependent extinction (Cowling et al., 2015; Galley et al., 2009; Schwery et al., 2015). Thus, it is not surprising that western Irano‐Turanian floristic region harbors approximately 27,000 species, and up to 40% of representatives that constitute this flora are endemic (Sales & Hedge, 2013; Takhtajan et al., 1986). This pattern can also be applied to *Sisymbrium*. Fourteen Sisymbrieae species (approximately 30% of the whole tribe) occur in the western part of the Irano‐Turanian floristic region, and half of them (sensu BrassiBase) are confined to this floristic region. Furthermore, ten individual speciation events within this area were inferred (third highest after Mediterranean) and the ancestral range reconstruction put the origin of this tribus somewhere into the Mediterranean and Irano‐Turanian floristic region (Figure 5), again supporting the hypothesis that this tribe (as well as the whole family) originated in the Irano‐Turanian floristic region.

Adjacent to the Irano‐Turanian floristic region is another biodiversity hotspot of the Old World—the Caucasus. Currently, there are around 7,500 plant taxa described from the Caucasus region, with 35% having a status of an endemic species (Gagnidze et al., 2002; Schatz et al., 2009), including four *Sisymbrium* taxa. Palaeontological, palaeoclimatical, and genetic data point out that the Caucasus region was one of the main glacial refugial centers for fauna and dendroflora during the Quaternary together with Iberian, Italian, and Balkan Peninsula (Hewitt, 2000, 2011; Stewart et al., 2010). Furthermore, intermontane basins and high mountain plateaus of the greater Caucasus region were also recognized as a cryptic southern refugium for dry‐ and cold‐adapted species during the interglacial periods (Fjellheim et al., 2006; Skrede et al., 2006). The exact extent of Pliocene and Pleistocene glaciations of the Caucasus is, however, unclear (Milanovsky, 2008; Mitchell & Westaway, 1999). Nevertheless, recent isotope dating studies and an overall high level of endemism in this region (Gagnidze et al., 2002; Nakhutsrisvili et al., 2017) suggest that the Caucasian glaciations were not as vast as previously thought. Pliocene to Pleistocene transition had a tremendous effect on the vegetation composition of the Greater Caucasus area. With the decrease in temperature and humidity, the thermophilic forests flora was gradually replaced by a more robust temperate dendroflora without a modern analogue (Klopotovskaya, 1973; Naidina & Richards, 2016). Gradual intensification of the continental climate and reduced humidity caused an expansion of forest steppes and steppes through the Greater Caucasus area. Palaeovegetational analyses dated this expansion to be not older than 1.8 MYA (Connor & Kvavadze, 2009; Joannin et al., 2010; Messager et al., 2013; Naidina & Richards, 2016; Tagieva et al., 2013). This coincides with *Sisymbrium* clade endemic to Caucasus region, which started to diverge around the same time and its most recent common ancestor that invaded this area through East European Plain in the Pliocene (Figures 4 and 5).

### 4.6 Biogeography of *Sisymbrium* in Eurasia

A peculiar relationship is observed between European *S. strictissimum* and its sister clade, consisting of East Asian *S. luteum* and *S. yunnanense*. All three species exhibit similar morphology (Vassilczenko, 1939; Zhou et al., 2001) and distinctive habitat preference. While most of the other *Sisymbrium* species are either widely distributed weeds or show a tendency toward open dry habitats, these three species can be found in more humid and predominantly shaded habitats in forests, thickets, ravines, or mountain slopes near water bodies (Brach & Song, 2006; Brandes, 1991). Assuming that the ecology of the last common ancestor of these species did not vary greatly, the expansion of the clade through the late Miocene/early Pliocene forests of central Asia is highly probable. During this era, most of Europe was covered in warm‐temperate evergreen broadleaved and mixed forest, Western and Central Asia (between 45°N and 53°N) in temperate deciduous broadleaf forests, while the coastline of Eastern Asia was dominated by similar forest types as present in Europe, which transitioned into temperate deciduous broadleaved savanna biome in the inland (Bezrukova et al., 2003; Haywood et al., 2002; Ivanov et al., 2011; Pound et al., 2012). According to our study, the diversification into two distinct contemporary eastern (*S. luteum* and *S. yunnanense*) and western lineage (*S. strictissimum*) took place around early Pleistocene (Figures 4 and 5). This coincides with the era when the continuous forest belt started to fall apart due to the increased aridity and lower temperatures (Demske et al., 2002; Frenzel, 1968; Velichko, 2005).

Despite their wide distribution range, the fast‐evolving ITS locus in *S. loeselii* and *S. officinale* remained monomorphic across the species’ whole distribution area. While the latter showed no ambiguous signals in either of the investigated loci, all *Sisymbrium loeselii* accessions had to be cloned to retrieve two unambiguous ITS copies. Because the multi‐copy ITS region is under strong influence of concerted evolution (Koch et al., 2003), the persisting two ITS copies might be an indicator of a recent hybridization event through which *S. loeselii* emerged. The young age is also supported by our time estimation analyses, placing the split between the two coexisting ITS copies into the western Euro‐Siberian steppe of the middle Pleistocene (Figures 4 and 5). Both species are nowadays distributed worldwide and can be commonly found near human settlements, along roadsides and hedges, or growing on heavily nitrogen contaminated dry and loamy soils ( Malyschev & Peschkova, 2004). Exhibiting a weed‐like ecology, these two species are excellent competitors that can successfully occupy wide ranges of disturbed habitats in a short period of time (Holzner & Numata, 2013). Therefore, quick and extensive repeated migrations through human migratory pathways might be accounted for the lack of geographically correlated genetic footprint even in the fast‐evolving ITS region. Nevertheless, sound conclusions on this issue can be drawn based only on extensive next‐generation sequencing datasets and proper sampling efforts.

In contrast to *S. loeselii* and *S. officinale*, some *Sisymbrium* species exhibit geographically correlated diverging ITS copies. Genetic splits found in widely distributed species were mostly of younger age indicating a potential influence of middle and late Pleistocene events. This era is characterized by an intensified development of permafrost and continental climate, major trans‐ as well as regressions of water bodies and major continental glaciations that got gradually reduced toward the end of the Pleistocene (Head et al., 2008; Head et al., 2008; Velichko, 2005). These parameters have shaped the evolutionary history also of other plant taxa, such as *Adonis* (Kropf et al., 2019), *Camelina* (Žerdoner Čalasan et al., 2019), *Linum* (Plenk et al., 2017), and *Schivereckia* (Friesen et al., 2020). This could likewise be the case in a two‐way split in *Sisymbrium altissimum*, *S. irio*, *S. volgense,* or three‐way split in predominantly Mediterranean *S. erysimioides*, all dated to the late or the end of middle Pleistocene. A slightly older split can be inferred from *Sisymbrium heteromallum* dating to the middle Pleistocene in the continental Asia, and an even older split between *S. polymorphum* accessions, dated to late Miocene/early Pliocene into Middle and Central Asia (Figures 4 and 5). The latter one is of great interest for two reasons. Firstly, *Sisymbrium polymorphum* is, as the epithet already suggests, morphologically as well as genetically an extremely variable species. This is also illustrated in our case, where *S. linifolium* is retrieved as a sister group to the eastern group of *S. polymorphum* s.l. accessions, rendering the *S. polymorphum* paraphyletic. This paraphyly also found in Chen et al., 2019, coupled with immense morphological variability of this species group, indicates a complex problem, which is out of the scope of this study. Secondly, *Sisymbrium polymorphum* is a widely distributed species found solely across the entire Eurasian steppe belt. Assuming that its ecology has not changed extensively since the past makes the species a suitable proxy to infer past florogenetic patterns of the Eurasian steppe belt. Molecular signals in typical steppe plant species reflect the climate‐landscape history of the steppe and thus allow for a finer resolution of the history of the steppe belt in comparison with floristic and fossil‐based methods. Overall, all these splits cannot be rigorously assigned to certain geological events due to a limited taxon sampling. Nevertheless, our study might serve as the starting point for other *Sisymbrium*‐specific studies that investigate different aspects of the onset and development of different Eurasian geographical entities.

### 4.7 Taxonomic update on *Sisymbrium*

In view of the above‐mentioned paraphyly of *Sisymbrium* that embeds the single species of *Ochthodium*, the latter genus is synonymized here with the prior one. Before molecular phylogenetic studies, proximity of the two genera has never been assumed due to considerable differences in the fruit and seed characters (many‐seeded dehiscent linear‐cylindric or conical siliques with smooth papery valves and seeds with incumbent cotyledons in *Sisymbrium* versus two‐seeded indehiscent ellipsoid to subglobose silicles with verrucose corky valves and seeds with accumbent cotyledons in *Ochthodium*) that were traditionally the key features in the systematics of the family. It is therefore understandable that Warwick et al. (2010), who first revealed the phylogenetic proximity of the two genera, plead for the additional study of *Ochthodium* and left it unassigned to a tribe instead of including it in Sisymbrieae. However, the rest of characters such as habit and life form (rosette‐forming biennial), indumentum of simple trichomes, leaf morphology (lyrate‐runcinate), flowers (yellow, middle‐sized, widely open) fit well the molecular signals. Hence, it is just another case of evolutionary recent drastic deviation of fruit character(s) not accompanied by serious genetic changes and alterations of other morphological features, a phenomenon known in various other groups of Brassicaceae, for example, *Twisselmannia* Al‐Shehbaz versus *Tropidocarpum* Hook. of Descurainieae Al‐Shehbaz et al., (Al‐Shehbaz, 2003), *Drabopsis* K. Koch versus *Draba* L. of Arabideae DC. (Al‐Shehbaz & Koch, 2003), several segregate genera versus *Heliophila* L. of Heliophileae DC. (Mummenhoff et al., 2005) or *Tchihatchewia* Boiss. versus *Hesperis* L. of Hesperideae DC. (German & Al‐Shehbaz, 2018), to name a couple of examples. Thus, the fact that in anthesis *Ochthodium* is indistinguishable from *Sisymbrium* is obviously not a coincidence.

***Sisymbrium*** L., Sp. Pl.: 657. 1753. = *Ochthodium* DC., in Mém. Mus. Hist. Nat. 7:236. 1821, syn. nov.

***Sisymbrium aegyptiacum*** (L.) German, Zerdoner & Al‐Shehbaz, comb. nov. ≡ *Bunias aegyptiaca* L., Syst. Nat., ed. 12, 3:231. 1768. ≡ *O. aegyptiacum* (L.) DC., Syst. Nat. [Candolle] 2:423. 1821.

## CONFLICT OF INTEREST

The authors declare no conflict of interest.

## AUTHOR CONTRIBUTIONS

**Anže Žerdoner Čalasan:** Conceptualization (equal); formal analysis (lead); investigation (lead); visualization (lead); writing – original draft (lead); writing – review and editing (lead). **Dmitry A. German:** Writing‐original draft (equal); writing – review and editing (equal). **Herbert Hurka:** Conceptualization (equal); funding acquisition (equal); writing – original draft (equal); writing – review and editing (equal). **Barbara Neuffer:** Conceptualization (equal); funding acquisition (equal); writing – original draft (equal); writing – review and editing (equal).

## ETHICAL APPROVAL

This article does not contain any studies with animals carried out by any of the authors.

## SAMPLING AND FIELD STUDIES

The study was performed in compliance with the Convention on Biological Diversity (CBD).

## Supporting information

Appendix S1

Appendix S2

Appendix S3

Appendix S4

Appendix S5

Appendix S6

Appendix S7

Supplementary Material

## Data Availability

DNA sequences: Genbank accessions MW270940–MW270994, MW271628–MW271779, MW280305–MW280306, MW281257–MW281310, MW319077–MW319132, MW319133–MW319187, MW345417–MW345468, MW345469–MW345520, MW355774–MW355817 & MW331329–MW331436.
