# Supplementary material for: A story from the Miocene: Clock‐dated phylogeny of Sisymbrium L. (Sisymbrieae, Brassicaceae)
Source: Ecol Evol. 2021 Mar 2;11(6):2573–95. doi: 10.1002/ece3.7217 (PMC7981217; doi:10.1002/ece3.7217)
Supplement: Supplementary file 7 — Appendix S7 [file ECE3-11-2573-s005.docx]

| NODE61: EVENT MATRIX:  Dispersal:2  Vicariance:0  Extinction:0  Event Route:  BE->BE^B^E->BE\|BE  PROBABILITY:  1.0000 | NODE62:  EVENT MATRIX:  Dispersal:2  Vicariance:0  Extinction:0  Event Route:  BE->BE^B^E->BE\|BE  PROBABILITY:  1.0000 |
| --- | --- |
| NODE63:  EVENT MATRIX:  Dispersal:2  Vicariance:0  Extinction:0  Event Route:  BE->BE^B^E->BE\|BE  PROBABILITY:  0.6006 | NODE64:  EVENT MATRIX:  Dispersal:0  Vicariance:0  Extinction:0  Event Route:  H->H^H->H\|H  PROBABILITY:  0.9982 |
| NODE65:  EVENT MATRIX:  Dispersal:0  Vicariance:0  Extinction:0  Event Route:  H->H^H->H\|H  PROBABILITY:  0.9835 | NODE66:  EVENT MATRIX:  Dispersal:1  Vicariance:1  Extinction:0  Event Route:  BH->BEH->H\|BE  PROBABILITY:  0.2985 |
| NODE67:  EVENT MATRIX:  Dispersal:0  Vicariance:0  Extinction:0  Event Route:  G->G^G->G\|G  PROBABILITY:  1.0000 | NODE68:  EVENT MATRIX:  Dispersal:0  Vicariance:0  Extinction:0  Event Route:  G->G^G->G\|G  PROBABILITY:  1.0000 |
| NODE69:  EVENT MATRIX:  Dispersal:0  Vicariance:0  Extinction:0  Event Route:  G->G^G->G\|G  PROBABILITY:  1.0000 | NODE70:  EVENT MATRIX:  Dispersal:2  Vicariance:0  Extinction:0  Event Route:  BD->BD^B^D->BD\|BD  PROBABILITY:  1.0000 |
| NODE71:  EVENT MATRIX:  Dispersal:2  Vicariance:1  Extinction:1  Event Route:  BC->B->BDG->BD\|G  PROBABILITY:  0.3991 | NODE72:  EVENT MATRIX:  Dispersal:2  Vicariance:0  Extinction:0  Event Route:  B->B^B->BCH^B->BC\|BH  PROBABILITY:  0.1203 |
| NODE73:  EVENT MATRIX:  Dispersal:2  Vicariance:0  Extinction:0  Event Route:  BE->BE^B^E->BE\|BE  PROBABILITY:  0.7936 | NODE74:  EVENT MATRIX:  Dispersal:1  Vicariance:0  Extinction:0  Event Route:  B->B^B->BE^B->BE\|B  PROBABILITY:  0.4042 |
| NODE75:  EVENT MATRIX:  Dispersal:0  Vicariance:0  Extinction:0  Event Route:  D->D^D->D\|D  PROBABILITY:  1.0000 | NODE76:  EVENT MATRIX:  Dispersal:0  Vicariance:1  Extinction:0  Event Route:  BD->B\|D  PROBABILITY:  0.7283 |
| NODE77:  EVENT MATRIX:  Dispersal:1  Vicariance:0  Extinction:0  Event Route:  B->B^B->BD^B->BD\|B  PROBABILITY:  0.4143 | NODE78:  EVENT MATRIX:  Dispersal:0  Vicariance:0  Extinction:0  Event Route:  F->F^F->F\|F  PROBABILITY:  1.0000 |
| NODE79:  EVENT MATRIX:  Dispersal:0  Vicariance:0  Extinction:0  Event Route:  F->F^F->F\|F  PROBABILITY:  1.0000 | NODE80:  EVENT MATRIX:  Dispersal:0  Vicariance:0  Extinction:0  Event Route:  F->F^F->F\|F  PROBABILITY:  1.0000 |
| NODE81:  EVENT MATRIX:  Dispersal:0  Vicariance:1  Extinction:0  Event Route:  BF->F\|B  PROBABILITY:  0.1794 | NODE82:  EVENT MATRIX:  Dispersal:0  Vicariance:0  Extinction:0  Event Route:  C->C^C->C\|C  PROBABILITY:  1.0000 |
| NODE83:  EVENT MATRIX:  Dispersal:0  Vicariance:0  Extinction:0  Event Route:  C->C^C->C\|C  PROBABILITY:  1.0000 | NODE84:  EVENT MATRIX:  Dispersal:1  Vicariance:0  Extinction:0  Event Route:  C->C^C->CE^C->C\|CE  PROBABILITY:  0.5742 |
| NODE85:  EVENT MATRIX:  Dispersal:0  Vicariance:0  Extinction:0  Event Route:  C->C^C->C\|C  PROBABILITY:  0.5742 | NODE86:  EVENT MATRIX:  Dispersal:0  Vicariance:0  Extinction:0  Event Route:  C->C^C->C\|C  PROBABILITY:  1.0000 |
| NODE87:  EVENT MATRIX:  Dispersal:0  Vicariance:0  Extinction:0  Event Route:  C->C^C->C\|C  PROBABILITY:  1.0000 | NODE88:  EVENT MATRIX:  Dispersal:0  Vicariance:0  Extinction:0  Event Route:  C->C^C->C\|C  PROBABILITY:  1.0000 |
| NODE89:  EVENT MATRIX:  Dispersal:0  Vicariance:0  Extinction:0  Event Route:  C->C^C->C\|C  PROBABILITY:  1.0000 | NODE90:  EVENT MATRIX:  Dispersal:1  Vicariance:0  Extinction:0  Event Route:  C->C^C->AC^C->AC\|C  PROBABILITY:  1.0000 |
| NODE91:  EVENT MATRIX:  Dispersal:0  Vicariance:0  Extinction:0  Event Route:  C->C^C->C\|C  PROBABILITY:  1.0000 | NODE92:  EVENT MATRIX:  Dispersal:0  Vicariance:0  Extinction:0  Event Route:  C->C^C->C\|C  PROBABILITY:  1.0000 |
| NODE93:  EVENT MATRIX:  Dispersal:2  Vicariance:0  Extinction:0  Event Route:  AC->AC^A^C->AC\|AC  PROBABILITY:  0.6763 | NODE94:  EVENT MATRIX:  Dispersal:2  Vicariance:0  Extinction:0  Event Route:  AC->AC^A^C->AC\|AC  PROBABILITY:  0.5143 |
| NODE95:  EVENT MATRIX:  Dispersal:3  Vicariance:0  Extinction:0  Event Route:  ABC->ABC^A^B^C->ABC\|ABC  PROBABILITY:  1.0000 | NODE96:  EVENT MATRIX:  Dispersal:3  Vicariance:0  Extinction:0  Event Route:  C->C^A^C->ABC^A^C->ABC\|AC  PROBABILITY:  0.4676 |
| NODE97:  EVENT MATRIX:  Dispersal:0  Vicariance:0  Extinction:0  Event Route:  C->C^C->C\|C  PROBABILITY:  0.5394 | NODE98:  EVENT MATRIX:  Dispersal:0  Vicariance:0  Extinction:0  Event Route:  C->C^C->C\|C  PROBABILITY:  0.8772 |
| NODE99:  EVENT MATRIX:  Dispersal:3  Vicariance:1  Extinction:0  Event Route:  C->BCF->C\|BF  PROBABILITY:  0.1424 | NODE100:  EVENT MATRIX:  Dispersal:0  Vicariance:1  Extinction:0  Event Route:  ABD->A\|BD  PROBABILITY:  1.0000 |
| NODE101:  EVENT MATRIX:  Dispersal:2  Vicariance:0  Extinction:0  Event Route:  B->B^B^D->BD^B^D->BD\|BD  PROBABILITY:  0.3683 | NODE102:  EVENT MATRIX:  Dispersal:1  Vicariance:0  Extinction:0  Event Route:  ABD->ABD^B->B\|ABD  PROBABILITY:  0.2422 |
| NODE103:  EVENT MATRIX:  Dispersal:0  Vicariance:0  Extinction:0  Event Route:  A->A^A->A\|A  PROBABILITY:  1.0000 | NODE104:  EVENT MATRIX:  Dispersal:0  Vicariance:0  Extinction:0  Event Route:  A->A^A->A\|A  PROBABILITY:  1.0000 |
| NODE105:  EVENT MATRIX:  Dispersal:2  Vicariance:0  Extinction:0  Event Route:  A->A^A->ABD^A->A\|ABD  PROBABILITY:  0.4169 | NODE106:  EVENT MATRIX:  Dispersal:0  Vicariance:1  Extinction:0  Event Route:  AC->C\|A  PROBABILITY:  0.3177 |
| NODE107:  EVENT MATRIX:  Dispersal:1  Vicariance:0  Extinction:0  Event Route:  A->A^A->AC^A->A\|AC  PROBABILITY:  0.6137 | NODE108:  EVENT MATRIX:  Dispersal:1  Vicariance:0  Extinction:0  Event Route:  AC->AC^A->AC\|A  PROBABILITY:  0.6137 |
| NODE109:  EVENT MATRIX:  Dispersal:1  Vicariance:0  Extinction:0  Event Route:  C->C^C->AC^C->C\|AC  PROBABILITY:  0.5127 | NODE110:  EVENT MATRIX:  Dispersal:0  Vicariance:0  Extinction:0  Event Route:  B->B^B->B\|B  PROBABILITY:  1.0000 |
| NODE111:  EVENT MATRIX:  Dispersal:0  Vicariance:1  Extinction:0  Event Route:  BC->B\|C  PROBABILITY:  0.2601 | NODE112:  EVENT MATRIX:  Dispersal:3  Vicariance:0  Extinction:0  Event Route:  C->C^C->ABCE^C->ACE\|BC  PROBABILITY:  0.1306 |
| NODE113:  EVENT MATRIX:  Dispersal:1  Vicariance:0  Extinction:0  Event Route:  C->C^C->AC^C->C\|AC  PROBABILITY:  0.0709 | NODE114:  EVENT MATRIX:  Dispersal:0  Vicariance:0  Extinction:0  Event Route:  C->C^C->C\|C  PROBABILITY:  0.1893 |
| NODE115:  EVENT MATRIX:  Dispersal:0  Vicariance:0  Extinction:0  Event Route:  C->C^C->C\|C  PROBABILITY:  1.0000 | NODE116:  EVENT MATRIX:  Dispersal:0  Vicariance:1  Extinction:0  Event Route:  AC->A\|C  PROBABILITY:  0.7129 |
| NODE117:  EVENT MATRIX:  Dispersal:1  Vicariance:0  Extinction:0  Event Route:  C->C^C->AC^C->AC\|C  PROBABILITY:  0.2057 | NODE118:  EVENT MATRIX:  Dispersal:0  Vicariance:0  Extinction:0  Event Route:  A->A^A->A\|A  PROBABILITY:  1.0000 |
| NODE119:  EVENT MATRIX:  Dispersal:0  Vicariance:1  Extinction:0  Event Route:  AC->A\|C  PROBABILITY:  0.0917 |  |
| ==================================== | ==================================== |
| Dispersal Between Areas:  A->B:1  A->C:1  A->D:1  B->C:1  B->D:3.5  B->E:1.5  B->G:0.5  B->H:1  C->A:7  C->B:3  C->D:0.5  C->E:2  C->F:1  C->G:0.5  H->E:0.5 | Speciation Within Areas:  A:10  B:12  C:23  D:3  E:4  F:3  G:3  H:2 |
| Dispersal Table:  from to within  A 3.00 7.00 10  B 7.50 4.00 12  C 14.00 2.00 23  D 0.00 5.00 3  E 0.00 4.00 4  F 0.00 1.00 3  G 0.00 1.00 3  H 0.50 1.00 2 | Global Cost:  Global Dispersal: 45  Global Vicariance: 10  Global Extinction: 1 |
| Model testing:  LnL numparams d e j AICc AICc_wt  DEC -108.3 3 0.0056 0.050 0.012 223 1.00  DEC+J -160.3 2 0.033 0.026 0 324.8 7.8e-23  DIVALIKE -163.4 3 0.024 0.0050 0.0052 333.2 1.2e-24  DIVALIKE+J -175.8 2 0.029 2.0e-08 0 355.9 1.4e-29  BAYAREALIKE -175.8 3 0.029 1.0e-12 1.0e-05 358.1 4.7e-30  BAYAREALIKE+J -126.3 2 0.014 0.13 0 256.8 4.7e-08 | |
