# Supplementary material for: A story from the Miocene: Clock‐dated phylogeny of Sisymbrium L. (Sisymbrieae, Brassicaceae)
Source: Ecol Evol. 2021 Mar 2;11(6):2573–95. doi: 10.1002/ece3.7217 (PMC7981217; doi:10.1002/ece3.7217)
Supplement: Supplementary file 8 — Supplementary Material [file ECE3-11-2573-s006.docx]

**Appendix Captions**

**Appendix S1:** Voucher information table with GenBank accession numbers of the newly generated sequences of different Sisymbrieae accessions.

**Appendix S2:** Primer specifications and PCR programmes used in this study.

**Appendix S3:** Species names and GenBank numbers of accessions used in different Lineage II datasets. The ITS accessions in bold were generated in the course of this study, while the rest of the accessions was downloaded directly from the taxonomically curated Brassicaceae database (https://brassibase.cos.uni-heidelberg.de).

**Appendix S4:** Alignment statistics of individual loci in Sisymbrieae and Lineage II datasets.

**Appendix S5:** Nuclear DNA-based phylogenetic tree of Sisymbrieae reconstructed with ASTRAL. Numbers on branches correspond to node-support values based on bootstrapping. Codes next to species names refer to the isolation codes from Appendix S1.

**Appendix S6:** Chloroplast DNA-based phylogenetic tree of Sisymbrieae reconstructed with ASTRAL. Numbers on branches correspond to node-support values based on bootstrapping. Codes next to species names refer to the isolation codes from Appendix S1.

**Appendix S7:** Ancestral range reconstruction statistics
